# Ion Conductance-Based Perfusability Assay of Vascular Vessel Models in Microfluidic Devices

**DOI:** 10.3390/mi12121491

**Published:** 2021-11-30

**Authors:** Rise Akasaka, Masashi Ozawa, Yuji Nashimoto, Kosuke Ino, Hitoshi Shiku

**Affiliations:** 1 Graduate School of Environmental Studies, Tohoku University, 6-6-11 Aramaki-aza Aoba, Aoba-ku, Sendai 980-8579, Japan; rise.akasaka.q6@dc.tohoku.ac.jp; 2 Graduate School of Engineering, Tohoku University, 6-6-11 Aramaki-aza Aoba, Aoba-ku, Sendai 980-8579, Japan; masashi.ozawa.r4@dc.tohoku.ac.jp (M.O.); yuji.nashimoto.d8@tohoku.ac.jp (Y.N.); 3 Frontier Research Institute for Interdisciplinary Sciences, Tohoku University, 6-3 Aramaki-aza Aoba, Aoba-ku, Sendai 980-8578, Japan

**Keywords:** vascular vessel model, ion conductance assay, electrochemical device, perfusability assay, microfluidic device

## Abstract

We present a novel methodology based on ion conductance to evaluate the perfusability of vascular vessels in microfluidic devices without microscopic imaging. The devices consisted of five channels, with the center channel filled with fibrin/collagen gel containing human umbilical vein endothelial cells (HUVECs). Fibroblasts were cultured in the other channels to improve the vascular network formation. To form vessel structures bridging the center channel, HUVEC monolayers were prepared on both side walls of the gel. During the culture, the HUVECs migrated from the monolayer and connected to the HUVECs in the gel, and vascular vessels formed, resulting in successful perfusion between the channels after culturing for 3–5 d. To evaluate perfusion without microscopic imaging, Ag/AgCl wires were inserted into the channels, and ion currents were obtained to measure the ion conductance between the channels separated by the HUVEC monolayers. As the HUVEC monolayers blocked the ion current flow, the ion currents were low before vessel formation. In contrast, ion currents increased after vessel formation because of creation of ion current paths. Thus, the observed ion currents were correlated with the perfusability of the vessels, indicating that they can be used as indicators of perfusion during vessel formation in microfluidic devices. The developed methodology will be used for drug screening using organs-on-a-chip containing vascular vessels.

## 1. Introduction

Vascular organs play a crucial role in the transport of nutrients and cancer metastasis, and vessel formation has been widely investigated in vitro. Therefore, in vitro vascular vessel models have been constructed using vascular endothelial cells [1] for drug screening, fabrication of three-dimensional organs for regenerative medicine, and cellular mechanism analysis. Tube formation assays have also been widely used as a simple model with vascular endothelial cells cultured on Matrigel [2]. To mimic in vivo environments, vascular endothelial cells are typically co-cultured with fibroblasts both two- and three-dimensionally [3,4], resulting in the successful formation of capillary networks with lumen structures. In addition, vascular models have been constructed in microfluidic devices to resemble in vivo environments and to arrange cells/extracellular matrices (ECMs). Such devices provide perusable capillary networks consisting of vascular endothelial cells [5], with blood vessels formed by vasculogenesis and angiogenesis [6]. The formation of new blood vessels, known as vasculogenesis, occurs early in the developmental stage of a vascular tissue; progenitor cells rearrange themselves to form a lumen, resulting in the formation of new capillary blood vessels. In angiogenesis, new blood vessels form from pre-existing vessels through vascular sprouting.

For the evaluation of vascular models, microscopic imaging and electrochemical analyses have often been reported. For example, microscopic methods, such as fluorescence imaging, can visualize biomarkers via immunostaining [7]. In electrochemical analyses, electrochemical probe and electrode-array devices have been applied to vascular cells and tissues to evaluate their various functions, such as respiration activity and the production of nitric oxides, gene expression, cellular permeability, and cell barrier functions [8]. Transepithelial/endothelial electrical resistance (TEER) measurement is extensively used to monitor and examine integrity of the endothelial cell barrier. In this assay, an endothelial monolayer is prepared on a semipermeable membrane, and a pair of electrodes is placed on the apical and basal areas to measure the electrical resistance across the cellular monolayer [9,10]. In addition to these targets, great attention has been paid to characterizing their perfusion, especially organ-on-a-chip fields [11,12], because perfusion indicates organ maturity and affects drug influence. To evaluate perfusability, microfluidic devices are typically utilized. For example, in one previous study [5], center channels were filled with fibrin/collagen gel, and vascular endothelial cells were seeded on both sides of the gel. During culture, the cells migrated to the gel and formed vessel structures. In the same study, to evaluate the perfusion between channels, fluorescence tracers were introduced from upstream to downstream. In another simple method, cellular debris was also utilized to evaluate perfusion. Although perfusability can be successfully monitored using these methods, fluorescence tracers must be added to the culture medium. In addition, for real-time assays, a microscope must be set within a CO_2_ incubator. To overcome these methodological limitations, we present a novel strategy using ion currents to indicate the ion conductance between the channels of a microfluidic device.

**Figure 1:**
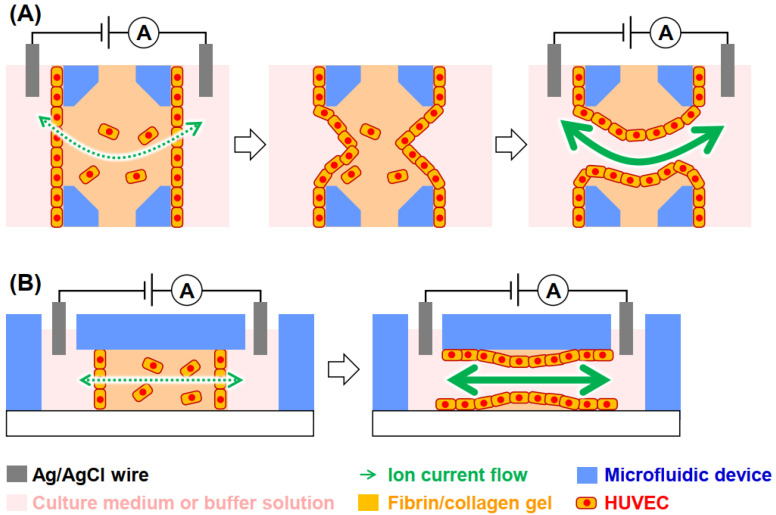
Strategy of ion conductance-based perfusability assay of vessel formation in microfluidic devices. (**A**) Top and (**B**) side views. Although the illustration is simplified, a potentiostat was used to measure ion currents.

Ion currents have been widely used to monitor various biosamples, such as cells [13], bacteria, viruses, peptides [14], and DNAs [15,16] passing through nano/micropores. In addition to nanopores based on biomaterials [17], solid-state nanopores have also been employed [18]. The detection mechanism is based on the increase in the electrical resistance of the pore in response to the excluded volume resulting from the introduced objects when applying electrical voltages. In scanning ion conductance microscopy, the ion conductance at a nanocapillary is used to measure the height of a sample [19,20]. In the present study, ion currents between channels separated by endothelial monolayers were monitored to evaluate their perfusion during vessel formation. A general outline of this strategy is shown in Figure 1. First, vascular vessel models were fabricated using human umbilical vein endothelial cells (HUVECs) in microfluidic devices. This model consists of a fibrin/collagen-gel channel containing HUVECs, HUVEC monolayers on both side walls of the gels, and channels of culture medium or measurement buffers. A pair of electrodes was inserted into the solutions, and potential was applied to measure the ion currents passing through the channels. The ion current is initially low because it is blocked by the endothelial monolayer, while higher ion currents are measured after vessel formation. As a proof-of-concept, we investigated the relationship between ion currents and perfusability during vessel formation. This strategy will be used for drug screening using organs-on-chip containing vessel structures.

## 2. Materials and Methods

### 2.1. Cell Culture

Green fluorescent protein (GFP) or red fluorescent protein (RFP)-expressing HUVECs (Angio-Proteomie, Boston, MA, USA) were cultured in endothelial cell growth medium 2 (ECGM2, PromoCell, Heidelberg, Germany) containing 1% penicillin/streptomycin (Gibco, Grand Island, NY, USA). Human lung fibroblasts (hLFs, Lonza, Basel Stücki, Switzerland) were cultured in fibroblast growth medium 2 (FGM-2, Lonza) containing 1% penicillin/streptomycin (Gibco). All cells were maintained in a humidified incubator at 37 °C with 5% CO_2_.

### 2.2. Microfluidic Devices

The microfluidic device consisted of polydimethylsiloxane (PDMS, Dow Toray Co., Ltd., Ishikawa, Japan) and a bottom cover glass (Matsunami, Osaka, Japan). PDMS microfluidic devices with a 100-µm channel height were fabricated using an SU-8 mold. Detailed information on the dimensions of the device is presented in Figure S1. Also see [21] for further details.

### 2.3. Vascular Vessel Models

**Figure 2:**
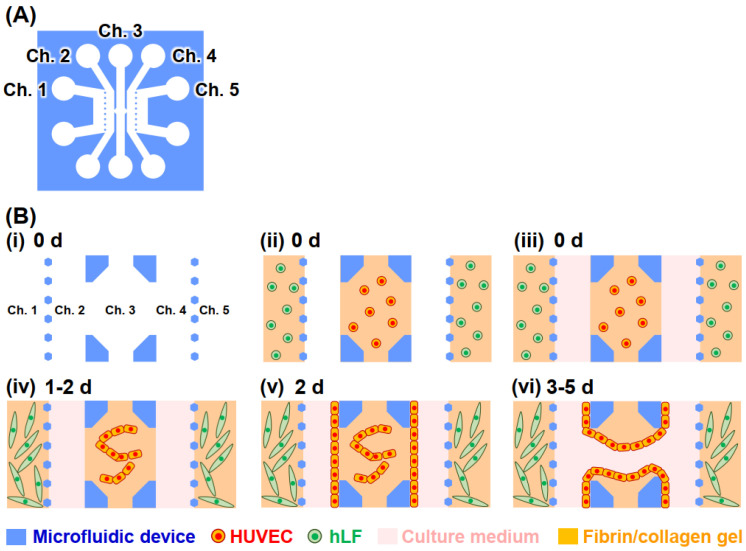
Fabrication of the vascular vessel models. (**A**) Device outline. (**B**) Cell culturing. (i)–(iv) HUVECs were cultured in gel in Ch. 3 for vasculogenesis. (v)–(vi) Additional HUVECs were seeded onto the gel sidewalls to prepare monolayers and were cultured for angiogenesis. Not to scale. Although only a simple vessel is illustrated here, in actuality a network of vessels is formed.

The fabrication process of the vascular vessel model is shown in Figure 2. The device consisted of five channels (Figure 2A). To set a bottleneck of ion current flows due to cells, PDMS bottlenecks were prepared, as shown in Figure S1. HUVECs (8.0 × 10^6^ cells/mL) were suspended in a precursor solution of fibrin/collagen gel (2.5 mg/mL fibrinogen (Sigma-Aldrich, Burlington, MA, USA), 0.15 U/mL aprotinin (Sigma-Aldrich), 0.2 mg/mL collagen (Corning Inc., Corning, NY, USA), and 0.5 U/mL thrombin (Sigma-Aldrich), and Dulbecco’s phosphate-buffered saline (D-PBS, Nacalai, Japan)) and the solution was then introduced into Channel (Ch.) 3 (Figure 2B). Owing to the surface tension, only Ch. 3 was filled with the solution. In addition, hLFs (5.0 × 10^6^ cells/mL) were suspended in the precursor solutions, and the solutions were introduced into Chs. 1 and 5. The solutions were incubated at 37 °C for 15 min to allow gelation. Then, Chs. 2 and 4 were filled with the culture medium of ECGM2, and these cells were cultured for 2 d to induce vasculogenesis at Ch. 3. After culturing for 2 d, the device was maintained in a tilted position, and additional HUVECs (5.0 × 10^6^ cells/mL) were introduced from Ch. 2 to be seeded on the side wall of the gel at Ch. 3. After 1 h to allow cell attachment, additional HUVECs (5.0 × 10^6^ cells/mL) were introduced from Ch. 4 to prepare endothelial monolayers on the opposite side of the gel. The cells were then cultured to induce angiogenesis, and the culture medium was changed daily, when cellular debris was observed to judge the perfusability of the channel separated by the fibrin/collagen gel. The network formation of HUVECs was subsequently observed under a fluorescence microscope (Eclipse Ts2, Nikon, Tokyo, Japan).

### 2.4. Ion Conductance Assay for Evaluation of the Perfusability of Vascular Vessel Models

The culture medium was replaced with PBS, and Ag/AgCl wires were then inserted into the solutions at Chs. 2 and 4, respectively. The wires were connected to a potentiostat (Figure S2), and the ion currents were measured using chronoamperometry and voltammetry. For the chronoamperometry, the potential was stepped from 0 to 0.2 V to obtain the ion currents. Current values over 50 s after the potential step were used for data visualization. For the voltammetry, potential was scanned from −1.0 to 3.0 V at 20 mV/s, and the current values at 1.0 V were used for visualization.

## 3. Results and Discussion

### 3.1. Vascular Vessel Models

**Figure 3:**
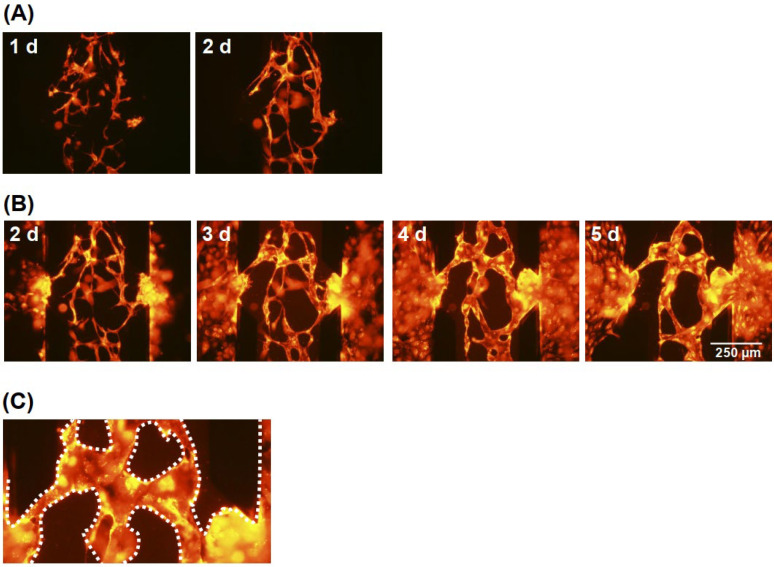
Fluorescence images of vascular vessel formation (**A**) before and (**B**) after preparation of the HUVECs monolayers. (**C**) Magnified image after 4 d. White dotted lines indicate the vessels.

When only the fibrin/collagen gel was prepared in Ch. 3, vessel structures were not constructed between the channels after 5 d. Therefore, HUVECs were incorporated into the gel to induce vascular anastomosis based on angiogenesis and vasculogenesis [22]. In addition, hLFs were cultured in a fibrin/collagen gel in Chs. 1 and 5 to improve the network formation [5]. Figure 3 shows fluorescence images of the HUVEC in the microfluidic devices. The HUVECs formed endothelial networks consisting of narrow cell fibers in the gels after 1 d and the cell fibers became thick after 2 d, although they remained relatively immature (Figure 3A). After culturing for 2 d, additional HUVECs were introduced into the channels, and initial HUVEC monolayers were successfully formed on both side walls of the gels (Figure 3B). Although there is no conclusive evidence supporting the formation of a monolayer, the term “monolayer” is used since HUVECs usually tend to form a monolayer after being cultured two-dimensionally. After 3 d, the HUVEC monolayers were connected to the HUVEC networks. After further culturing, vessel structures were formed and bridged between Chs. 2 and 4 (Figure 3C). Although the perfusability differed in every experiment due to slight variations in cell conditions and gel, perfusion was observed between Chs. 2 and 4 after 3–5 d. Although employing a different configuration, perfusable capillary networks were also observed to form within 3–4 d in the previous study [5].

### 3.2. Ion Conductance Assay for Evaluating the Perfusability of Vascular Vessel Models

**Figure 4:**
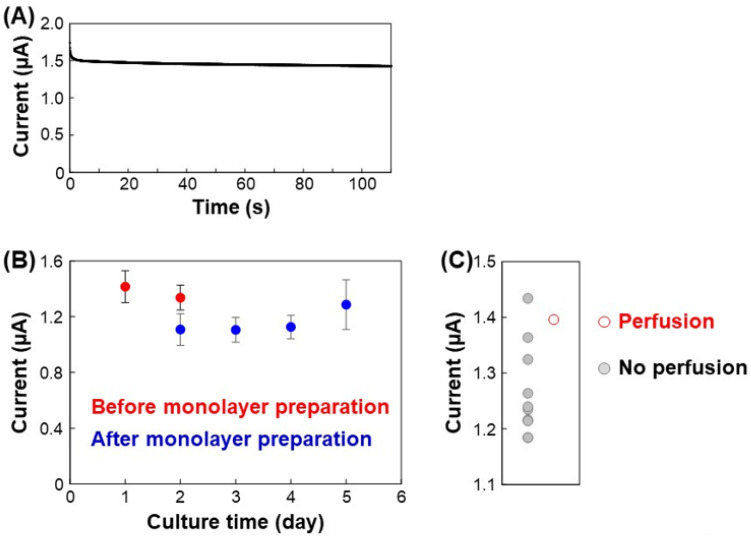
Ion currents at 0.2 V using PBS in Chs. 2 and 4 during the assay. (**A**) Amperogram after 1 d. (**B**) Ion current vs. culture time. The ion currents were obtained from the amperograms at 0.2 V. N = 10. The error bars indicate SDs. (**C**) Ion currents and perfusability after 5 d. The current value of the perfusion sample was higher than those of seven out of the eight samples with no perfusion.

Amperometry was conducted at 0.2 V to obtain ion current values. As we used 0.2 V during our scanning ion conductance microscopy, the potential was selected first. Without HUVECs in the device, the ion currents were approximately 1.4 µA. The ion currents depend on the solution resistance and the size of the PDMS bottleneck as shown in Figure S1. Figure 4A shows an amperogram after 1 d. The amperogram shows the short-term response. Although the ion currents decreased slightly, they were generally stable. When HUVECs were incorporated into the gel in Chs. 3 and cultured for 1 d, the ion current was 1.42 ± 0.11 µA, which was similar to the condition without any cells (Figure 4B). This indicates that the HUVECs in the fibrin/collagen gels did not reach and cover the PDMS bottleneck after 1 d. In contrast, when the initial HUVEC monolayers formed on the PDMS bottleneck after 2 d, the ion currents decreased to 1.11 ± 0.08 µA because the monolayers blocked the ion current flow. As the monolayers were immature at this stage and because there were gaps between the cells, the ion currents were not completely blocked. The ion currents then gradually increased, reaching 1.29 ± 0.09 µA after 5 d, indicating that thick vessel structures had formed between the channels, which is consistent with the fluorescence images. Perfusion of cellular debris from Chs. 2 to 4 was observed in one sample after 5 d, and the ion current was relatively high (Figure 4C). Although cellular debris flow was not observed in other samples, the ion currents increased during the culturing period, indicating that the vessels might penetrate the gel channel but the lumen size might remain insufficient to allow the movement of cellular debris. Based on these observations, vessel formation and perfusability were successfully evaluated based on the ion conductance between the device channels. To change largely current values before and after vessel formations, it is necessary to optimize the channel width. If the solution resistance in areas outside Ch. 3 is reduced by enlarging the width and height, the performance of the sensing will be improved. Also, the increase of the PDMS bottleneck will be effective.

**Figure 5:**
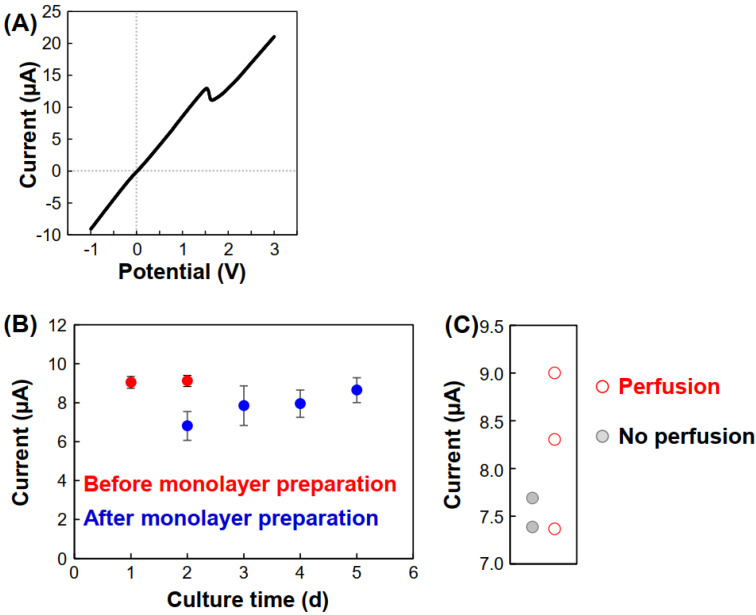
Ion currents at 1.0 V using PBS in Chs. 2 and 4 during the assay. (**A**) Current vs. potential after 1 d. (**B**) Ion current vs. culture time. The values were obtained from the currents 1.0 V of the I-V curves. N = 5. The error bars indicate SDs. (**C**) Ion currents and perfusability after 4 d. The current values of two out of the three perfusion samples were higher than those of the samples with no perfusion.

Subsequently, current-voltage (I-V) curves were obtained to characterize the ion currents (Figure 5A). As the currents depended on the resistance between the channels, the current increased linearly from −1.0 to approximately 1.5 V, but a peak was observed at approximately 1.5 V. This indicates that currents above 1.5 V do not reflect ion conductance but, rather, the electrochemical reaction at the Ag/AgCl wires. Because the peak potentials slightly differed in each experiment, the currents at 1.0 V were used in a graph of the currents with culture time (Figure 5B). The resulting graph shape is similar to that produced using the amperogram at 0.2 V, indicating that 1.0 V might be suitable for analysis because of the large current values, and that the potential did not have any significant toxic effect on vessel formation. Also, there is a strong correlation between the perfusability and the current values at 1.0 V (Figure 5C). However, unfortunately, S/N was not significantly improved using this 1.0 V value in this experimental condition.

**Figure 6:**
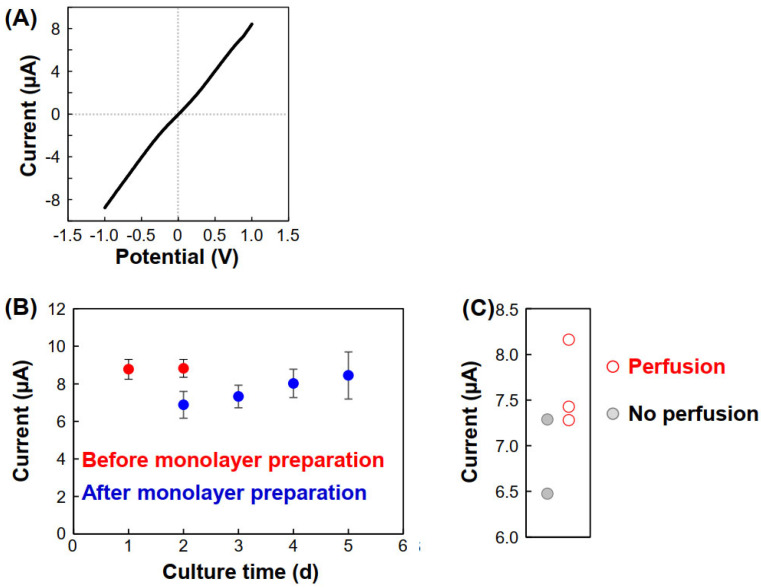
Ion currents at 1.0 V using the culture medium in Chs. 2 and 4 during the assay. (**A**) Current vs. potential after 1 d. The data is shown from -1.0 to 1.0 V. (**B**) Ion current vs. culture time. The values were obtained from the currents at 1.0 V of the I-V curves. N = 4–5. The error bars indicate SDs. (**C**) Ion currents and perfusability after 3 d. The current values of two out of the three perfusion samples were higher than those of the samples with no perfusion.

In previous experiments, the culture medium was changed to PBS during the assay given its simple components; however, this is time-consuming, and medium changes are not suitable for real-time analysis. Therefore, the culture medium was used instead of PBS. As shown in the I-V curves in Figure 6A, the ion currents were similar to those obtained using PBS, indicating that changing the culture medium to a simple buffer is not necessary. Indeed, Figure 6B,C show that perfusion was successfully evaluated.

Electrochemical approaches have been applied to evaluate vascular models. For example, Wong et al. [23,24] reported a novel on-chip microfluidic permeability assay using an electroactive tracer instead of traditional fluorescent tracers. Their method used a bilayer microfluidic device containing a porous membrane or ECM gel. Monitoring redox compounds diffused to the bottom from the top through an endothelial monolayer. In these previous studies, an endothelial barrier based on cell–cell junctions was successfully evaluated. In contrast, our study provides a novel electrochemical methodology for evaluating perfusability. To the best of our knowledge, this is the first report of an ion-conductance-based perfusability assay for vascular vessel models.

## 4. Conclusions

We have presented a novel methodology for evaluating vascular formation in microfluidic devices in which ion currents are monitored through the HUVEC monolayers. When vessels form from the monolayers and bridge the channels, the ion current increases. Because ion currents depend on perfusability, current values can be effectively used as indicators of perfusion. As our assay can be performed without microscopic imaging and the addition of tracers, it has significant advantages over conventional assays. In particular, given that it is difficult to observe the inside of the spheroids under bright-field imaging, our method should prove useful for the evaluation of vessel formation in cancer spheroids in chips [25]. In contrast, our assay cannot perform imaging. Nevertheless, electrode array devices, such as CMOS-based electrochemical devices [26], can address this problem. Furthermore, while we did not perform a real-time assay over an extended period of time, our electrochemical approach can be applied for this purpose. Although we fabricated complex networks, to simplify these models, a single-vessel microfluidic device needs to be developed. In the future, this technique will be applied for drug screening using organ-on-a-chip models.

## Supplementary Materials

The following are available online at https://www.mdpi.com/article/10.3390/mi12121491/s1, Figure S1: Device dimensions, Figure S2: Device during the measurements.
