# Supplementary material for: Ion Conductance-Based Perfusability Assay of Vascular Vessel Models in Microfluidic Devices
Source: Micromachines (Basel). 2021 Nov 30;12(12):1491. doi: 10.3390/mi12121491 (PMC8705798; doi:10.3390/mi12121491)
Supplement: Supplementary file 1 [file micromachines-12-01491-s001.zip › micromachines-1476676-supplementary.pdf]

Supporting information for

Ion conductance-based perfusability assay of vascular vessel models in microfluidic devices

Rise Akasaka[1], Masashi Ozawa[2], Yuji Nashimoto[2,3], Kosuke Ino[2,*] Hitoshi Shiku[2,*]

[1] Graduate School of Environmental Studies, Tohoku University, 6-6-11 Aramaki-aza Aoba, Aoba-ku, Sendai 980-8579, Japan

[2] Graduate School of Engineering, Tohoku University, 6-6-11 Aramaki-aza Aoba, Aoba-ku, Sendai 980-8579, Japan

[3] Frontier Research Institute for Interdisciplinary Sciences, Tohoku University, 6-3 Aramaki-aza Aoba, Aoba-ku, Sendai 980-8578, Japan

Corresponding authors: Kosuke Ino (kosuke.ino@tohoku.ac.jp) and Hitoshi Shiku (hitoshi.shiku.c3@tohoku.ac.jp)

Table of Contents

- Figure S1
- Figure S2

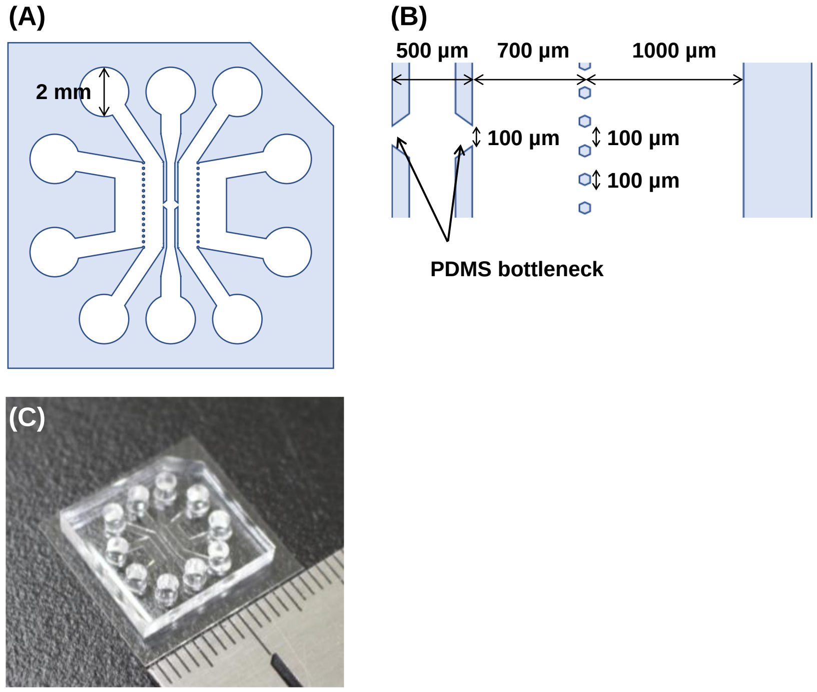

Figure S1

Device dimensions. (A) Overview. (B) Detailed information. The heights of all the channels were 100 μm. (C) Device photograph.

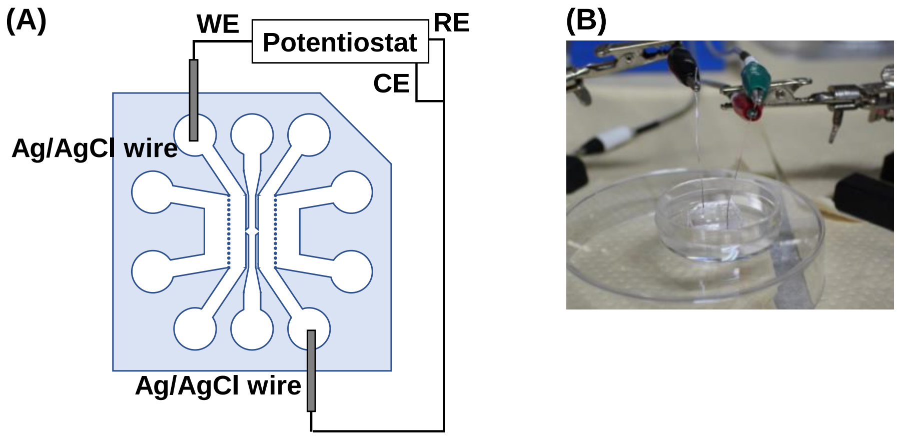

Figure S2

Device during the measurements. (A) Illustration. WE: working electrode. CE: counter electrode. RE: reference electrode. (B) Photograph.
